# Hemin-binding DNA structures on the surface of bacteria promote extracellular electron transfer

**DOI:** 10.1093/nar/gkaf790

**Published:** 2025-08-21

**Authors:** Obinna M Ajunwa, Gabriel Antonio S Minero, Sissel D Jensen, Rikke L Meyer

**Affiliations:** Interdisciplinary Nanoscience Center (iNANO), Aarhus University, Gustav Wieds Vej 14, 8000 Aarhus, Denmark; Center for Electromicrobiology, Department of Biology, Aarhus University, Ny Munkegade 114, DK-8000 Aarhus C, Denmark; Interdisciplinary Nanoscience Center (iNANO), Aarhus University, Gustav Wieds Vej 14, 8000 Aarhus, Denmark; Interdisciplinary Nanoscience Center (iNANO), Aarhus University, Gustav Wieds Vej 14, 8000 Aarhus, Denmark; Interdisciplinary Nanoscience Center (iNANO), Aarhus University, Gustav Wieds Vej 14, 8000 Aarhus, Denmark; Department of Biology, Aarhus University, Ny Munkegade 114, 8000 Aarhus, Denmark

## Abstract

Non-canonical DNA structures have been recently identified in bacterial biofilms, but their functional roles remain poorly understood. Here, we demonstrate that G-quadruplex (G4) DNA structures complexed with hemin enable extracellular electron transfer (EET) in biofilms. Using *Staphylococcusepidermidis* as a model organism, we show that extracellular DNA and hemin are essential for EET, with surface-associated G4-DNA/hemin complexes transferring electrons from bacteria to electrodes under anoxic conditions. Adding G4-DNA and hemin to growing biofilms promoted stable EET for days, demonstrating that these complexes serve as robust electrical conduits. The structural properties of G4-DNA, with its stacked guanine quartets facilitating π–π interactions with hemin’s porphyrin ring, create an effective electron transfer pathway. Additionally, the G4-DNA/hemin complex functions as a peroxidase-like DNAzyme, transferring electrons from bacteria to H_2_O_2_. This study reveals a previously unknown functional role for G4-DNA structures in biofilms, establishing them as components of bacterial EET. Our findings provide new insights into how non-canonical DNA structures contribute to bacterial energy conservation under oxygen limitation, and potentially also to their defense against oxidative stress during infection.

## Graphical Abstract

**Graphical Abstract:**
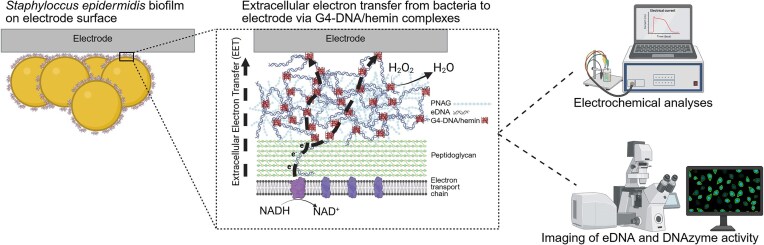

## Introduction

Nucleic acids are a common extracellular matrix component in bacterial biofilms. Recent research has discovered that extracellular DNA and RNA (eDNA and eRNA) in biofilms are not only in the canonical B-DNA double helix form. It can contain a variety of non-canonical structures, including Z-DNA, i-motif, and G-quadruplex DNA (G4-DNA) structures [1–4] forming in bundles of DNA strands that interconnect in net-like superstructures. Despite their apparent abundance, the functional roles of non-canonical structures in biofilms remain largely unexplored.

Bacterial biofilms can exhibit electroactivity, characterized by extracellular electron transfer (EET), which enables bacteria to move electrons to or from metabolic processes in the cell [5, 6]. This concept was previously thought to occur in a few prokaryotes that contain organelles or produce specific redox mediators [7]. However, recent findings suggest that EET might be widespread [8]. EET is particularly beneficial for bacteria that use solid electron acceptors or that live in steep chemical gradients where soluble electron acceptors are out of reach. Biofilms create such gradients by consuming oxygen, and bacteria in the anoxic layers can benefit from using EET to transfer electrons to the oxic layer. Recently, eDNA was implicated in EET in *Pseudomonas aeruginosa* biofilms. Electrons were transferred from bacteria in the anoxic part of the biofilm to oxygen at the biofilm surface via eDNA and small DNA-binding, redox-active molecules called phenazines: pyocyanin and phenazine carboxamide [9]. Pyocyanin production is unique to *P. aeruginosa*, and we wondered if a similar but more generic principle exists in other bacteria. With the discovery of G4-DNA and -RNA in bacterial biofilms, we propose that these non-canonical nucleic acid structures can play such a role through their unique ability to bind redox-active molecules.

It is well known that G4-DNA structures form a stable complex with hemin (Fe^3+^-protoporphyrin IX)—a highly abundant electron transfer molecule in biology. This interaction is facilitated by the planar guanine quartets of G4-DNA, which have increased potential for π-stacking and electron orbital sharing [10]. G4-DNA/hemin interactions are also reinforced by the co-ordination between Fe^3+^ in hemin with a nitrogen from G4-DNA or nearby water molecule [11]. The negatively charged DNA backbone also interacts with the hydrophobic porphyrin ring of hemin, contributing to the formation of a stable structure. As this interaction occurs, hemin bound to G4-DNA creates a planar stack on top of G4-DNA, forming a G4-DNA/hemin complex and enhances electron flow [12]. When stacks of G4-DNA are involved, this could be analogous to conductive molecular wires [13, 14]. In the presence of electron donors, the G4-DNA/hemin engages in redox cycling between electron donors, which reduce hemin’s Fe^3+^ to Fe^2+^, creating efficient electron movement. G4-DNA/hemin complex can engage in a peroxidase-like reaction, which can move electrons from an available electron donor to nearby peroxides functioning as electron acceptors [11]. This makes G4-DNA/hemin a functional DNAzyme with oxidoreductase activity.

Since the oxidoreductase properties of G4-DNA/hemin complexes are simply based on electron transfer, we hypothesize that G4-DNA/hemin complexes can facilitate EET in biofilms, and that this provides a generic concept for eDNA-mediated EET. This study aimed to demonstrate proof of concept for G4-DNA/hemin-mediated EET in bacteria and its role in enhancing EET in biofilms. We chose to work with *Staphylococcus epidermidis* as a model organism in this study due to its clinical relevance and because it was previously reported to produce large amounts of eDNA in the biofilm matrix—including G4-DNA structures [2].

In this work, we show that G4-DNA/hemin could interact with bacteria and facilitate EET. We then characterized the electrochemical signatures and charge transfer mediated by G4-DNA/hemin associated with both planktonic bacteria and biofilms of *S. epidermidis*. Our results support a scenario by which G4-DNA/hemin complexes associate with eDNA and support bacterial metabolism through EET. These findings suggest that EET can provide a mechanism for bacteria within biofilms to maintain metabolic activity through long-range electron transfer to oxygen or hydrogen peroxide in the infectious microenvironment and beyond.

## Materials and methods

### Chemicals and fluorescent stains

All chemicals used were first made as stock solutions. Hemin (Merck^®^) and protoporphyrin IX (Merck^®^) were prepared as 10 mM stock solutions in 200 mM Tris, 30% DMSO, and 100 mM NaOH (Merck®) at pH 11, and protected from light. Tris (Tris hydroxymethyl amino methane, pH 7.5, Merck^®^), modified MES buffer [25 mM MES (Sigma, M3671), 0.2 M NaCl (Sigma, S5886), and 0.010 M KCl (Sigma, 1.04936)], and DMSO stocks were prepared with filter-sterilized deionized water. Salts and sugars; NaCl, KCl, and xylose were prepared as sterile filtered 1 M stocks for buffer and culture preparations. Acetic acid (30% v/v) was used for pH adjustment of Tris buffers when prepared. Annealing buffer for G4-DNA was prepared by mixing 10 mM Tris, with 100 mM KCl (pH 7.2) and adjusting to pH 6.5 with acetic acid. Tyramide reagent was prepared by adding tyramide conjugated with Alexa Fluor 488 (1:100) (Invitrogen, B40953), 2 mM ATP (Thermo Scientific, R0441), and 0.1% hydrogen peroxide (Sigma, 216763) in modified MES buffer.

DNA-binding stain SYTO^TM^ 60 (20 μM working concentration) was prepared as 100× aliquots in sterile deionized water before use. A lipophilic dye FM 4-64 was prepared by mixing sterile distilled water to 1 mg/ml stock concentration. Working concentration for FM-464 was 10 μg/ml and aliquots for stains were made before storage at −20°C.

### Synthetic DNA, annealing, and characterization conditions

Oligonucleotides of guanine quadruplex (G4-DNA) sequences were purchased from Integrated DNA Technologies, Belgium, as 100 μM stock in 1 mM Tris-EDTA buffer. We used a monomer and a trimer of the c-Myc oncogene known to form G-quadruplex structures: c-Myc1: 5′- GA**GGGTGGGTAGGGTGGG**CGTCAACAGACTCGA-3′ and c-Myc3: 5′-GA**GGGTGGGTAGGGTGGGGAGGGTGGGTAGGGTGGGGAGGGTGGGTAGGGTGG**GCGTCAACAGACTCGA-3′. The part of the sequence that forms G-quadruplex is highlighted in bold. DNA annealing of oligonucleotides was done in an annealing buffer with composition stated above. Annealing was done by preparing 100 μl of 50 μM oligonucleotide stock in annealing buffer (pH 6.5), heating to 90°C for 3 min, and gradual cooling to 30°C. The structure of annealed oligonucleotides was confirmed by recording the circular dichroism (CD) spectrum using a J-810 spectropolarimeter at 220–320 nm, scanning speed 50 nm/min, and 2 nm bandwidth, and ultraviolet/visible spectrophotometer (UV/vis) also at 220–320 nm. For CD and UV/vis measurements, 5 μM oligonucleotides were prepared from sub-stocks, and 70 μl sample was loaded into the spectropolarimeter and spectrophotometer, respectively. CD signals were converted to ellipticity Δϵ (M^−1^ cm^−1^) using Beer–Lambert’s law and recorded.

### Strains, cultures, and media

Tryptic soy broth medium (TSB, Merck) and Brain heart infusion medium (BHI, Merck) were prepared according to manufacturers’ specifications and used for culture preparations. Solid agar plates were prepared by mixing broth medium with 15 g/l agar powder. Autoclaved media broths supplemented with an additional 200 mM NaCl were used for growing bacteria in overnight cultures. Bacterial cultures were made as overnight cultures by inoculating from single colonies on agar into 20 ml BHI/NaCl or TSB/NaCl broth depending on culture requirements, and incubated for 15–20 h at 37°C with constant agitation at 150 rpm. *Staphylococcus epidermidis* 1457, *S. epidermidis* 1457 Δ*atlE*, *S. epidermidis* 1585 pTX*icaADBC* were used in this study [15–18]. Strains were stored at −80°C as stock cultures in cryotubes in 30% glycerol. For experiments with hemin, TSB/NaCl medium was supplemented with 5 μM hemin unless stated otherwise.

### Visualization of G4-DNA structures by immunolabeling

Fluorescence-conjugated antibodies were used for immunolabeling of DNA structures. G4-DNA specific antibody BG4 (goat monoclonal IgG lambda, Ab00174-24.1) with 0.02% proclin was purchased as 1 mg/ml preparations in phosphate-buffered saline (PBS) from Absolute Antibodies^®^ with the fluorescent label Atto 488 and stored at 4°C. B-DNA specific antibody (mouse monoclonal, AB27156) was purchased from Abcam^®^ without a fluorescent label. An anti-mouse antibody (goat monoclonal IgG H + L, Cat. No.: A-31553, Invitrogen^®^), labelled with Alexa Fluor 405 was purchased as 2 mg/ml preparation in PBS with 0.05% sodium azide and 50% glycerol, and stored at −20°C, and used as a secondary antibody for B-DNA detection (AB2). Both antibodies were mixed with 3% bovine serum albumin (BSA) in PBS, which served as a blocking agent for non-specific binding during immunolabeling experiments. Planktonic cultures were suspended for 2 min in 3% BSA in PBS before addition of BG4 antibody (working concentration 1:100 in 3% BSA in PBS) and incubation for 90 min at room temperature. Immunolabeled bacteria were then briefly washed in 100 mM KCl and transferred to glass slides for microscopy.

For biofilm samples, the medium was removed, and biofilms were gently washed with 3% BSA in PBS before adding 60 μl BG4 antibody (1:100) in 3% BSA in PBS and incubating for 90 min with gentle shaking at 50 rpm at room temperature. Without washing or decanting, 60 μl AB1 (1:100) in 3% BSA was added, and samples were incubated for another 90 min under the same conditions. For the next step, biofilms were gently washed with 3% BSA before the addition of 60 μl of the secondary antibody AB2 (1:100) in 3% BSA in PBS and 60 min incubation. Finally, biofilms were washed with 3% BSA in PBS and stained with 10 μg/ml FM-464 solution in 100 mM NaCl to visualize the bacteria.

CLSM was performed using a LSM700 microscope (Carl Zeiss) equipped with a 63× objective (Plan-Apochromat, NA1.4 oil immersion objective). We used the brightfield transmission to see unstained bacteria, 488 nm excitation and >660 nm emission to visualize FM4-64 stained bacteria, 488 nm excitation and 520–600 nm emission for the Atto-488 conjugated BG4 antibody, and 405 excitation and 410–477 nm emission for the Alexa Fluor 405-conjugated AB2 antibody.

### Establishment of an experimental model with high or low amounts of G4-DNA

To investigate the contribution of G4-DNA to EET, we first established an experimental model in which bacteria contained many or few G4/hemin complexes associated with the bacterial surface. We hypothesized that G4-DNA would associate with extracellular polysaccharides and therefore acquired a strain in which polysaccharide production could be controlled via an inducible promoter. *Staphylococcus epidermidis* 1585 pTX*icaADBC* was grown overnight in BHI/NaCl supplemented with tetracycline (20 μg/ml), and xylose (2% w/v) to induce production of poly-n-acetyl glucosamine (PNAG). Bacteria grown in the absence of xylose were used as a polysaccharide-deficient control.

To prepare the experimental model, overnight culture of bacteria were collected in 1 ml broth and centrifuged (5 min at 906 × *g*), washed, and resuspended in 100 mM KCl to an optical density at 600 nm (OD_600_) of 0.5. Annealed G4-DNA (5 μM) and/or hemin (5 μM) was added (5 μM hemin best supported hemin tolerance by *S. epidermidis*), and samples were vortexed (1 min), incubated at room temperature for 1 h in the dark, centrifuged (2 min at 906 × *g*), and resuspended in 100 mM KCl to wash away unbound G4-DNA or hemin. Surface-bound G4-DNA was visualized by fluorescence immunolabeling as described above.

Annealed c-Myc1 and c-Myc3 G4-DNA sequences were adsorbed on PNAG producing *S. epidermidis* 1585 pTX*icaADBC* separately and immunolabelled for comparison. After confirming that *S. epidermidis* 1585 pTX*icaADBC* could adsorb G4-DNA to the bacterial surface when PNAG production was induced, this strain was used as a model organism with high or low amounts of G4-DNA structures in the subsequent experiments. All subsequent experiments were done with the G4-DNA sequence with highest binding.

Prior to the experimental model, concentrations of G4-DNA/hemin concentration tolerable to planktonic cells were determined and used for subsequent experiments with the *S. epidermidis* 1585 pTX*icaADBC* strain. BHI/NaCl broths supplemented with tetracycline (20 μg/ml), with and without xylose (2% w/v), were prepared to test the effect of PNAG production by planktonic cells on hemin tolerance. Overnight cultures were collected in 1 ml broths, centrifuged (5 min at 906 *× g*), and pellets were resuspended in same medium broths to an optical density (OD_600_) of 0.01 in 2 ml volumes before transferring to sterile 48-well plates (Sarstedt AG & Co). Different hemin concentrations (0, 5, 40, and 200 μM) ±5 μM, annealed c-Myc3 were then added. Unlike hemin, G4-DNA was not considered toxic, and concentration used (5 μM) in this experiment was kept constant. Experiments were carried out in triplicates and kept in the dark. Microtitre plates with culture were incubated for 24 h with shaking (100 rpm) at 37°C. After incubation, 0.1 ml of each of the cultures was serially diluted 10-fold in sterile deionized water. Dilution 10^−7^ was plated onto BHI agar by transferring 0.1 ml onto the agar. Inoculum were evenly spread on the agar using sterile cell spreader. Agar plates were incubated at 37°C for 24 h. Colonies were counted after incubation, and CFU/ml was determined. Logarithmic values of the CFU/ml were calculated and recorded.

### Electroanalysis of planktonic bacteria adsorbed to the electrode

*Staphylococcus epidermidis* 1585 pTX*icaADBC* was grown overnight in BHI/NaCl supplemented with tetracycline (20 μg/ml), and ± xylose (2% w/v) to induce polysaccharide production. Bacteria were washed by centrifugation (5 min at 1118 × *g*) and adjusted to OD_600_ 0.5 in 100 mM KCl with G4-DNA and/or hemin treatments as described earlier. For comparison, electroanalyses with planktonic *S. epidermidis* 1457 and *S. epidermidis* 1457 Δ*atlE* were also carried out on overnight cultures grown in BHI/NaCl, washed and suspended in 100 mM KCl to OD_600_ 0.5 with G4-DNA + hemin treatment as described earlier.

The SPEs (Metrohm DropSens DRP-ITO10-U20, Metrohm Spain) had a 4 mm diameter transparent Indium Tin Oxide (ITO) working electrode (WE) with surface area of 0.126 cm^2^, a graphite counter electrode, and Ag pseudo-reference electrode. Prior to use, screen printed electrodes (SPEs) were sterilized in 70% v/v ethanol, washed thrice in sterile deionized water, and air dried. Bacterial suspensions (100 ml) were then deposited on SPEs and left to adsorb for 15 min. During deposition, cells were made to cover the whole surface area of working, reference, and counter electrode. Excess cells were gently rinsed with 100 mM KCl and SPEs were subsequently connected to a computer-controlled multichannel PalmSens 4 potentiostat (PalmSens^®^ Netherlands) equipped with an EmStatMUX8-R2 multiplexer.

All electrochemistry data were collected and analysed using the PalmSens PsTrace 5.9 software. Cyclic voltammetry (CV) was run between −0.8 and 0.8 V with a step size of 0.01 V and a scan rate of 0.05 V/s for three scan cycles, and the last scan was selected per sample. Samples were run in triplicates and the averages were plotted. Differential pulse voltammetry (DPV) was carried out by first pretreating at 0.2 V for 2 min before running with −0.8 V as start potential and 0.8 V as end potential, with scan rate of 0.05 V/s, step size 0.01 V, pulse time 0.02 s, and current range from 10 nA to 1 mA. The average scans of three biological replicates were recorded for each voltammetric experiment.

Chronoamperometry (CA) was also carried out using 0.4 V poised potentials for 300 s after 200 s pre-treatment at same potential. Values of total charge was recorded as charge including charge generated during pretreatment and during analysis. Multi-step chronamperometry (MSCA) was carried out by switching between poised potentials 0.4 and −0.4 V for 30 s with 3 s between switches.

At the end of the experiment, the adsorbed bacteria and the presence of G4-DNA structures were visualized by immunolabeling and microscopy as described earlier. Comparative CV analyses were carried out on c-Myc1 and c-Myc3 G4DNA treated planktonic cells of polysaccharide producing *S. epidermidis* 1585 pTX*icaADBC* in the presence of hemin. G4-DNA with higher electroactivity was selected for further analyses.

### Blocking of electron transfer with G4-DNA-binding antibodies

We sought to validate the contribution of G4-DNA/hemin to electroactivity, as hemin could potentially also associate with other biomolecules on the bacterial surface. We therefore used a G4-DNA specific antibody to block the electron flow from G4-DNA/hemin on bacteria to the electrode. Overnight cultures of *S. epidermidis* 1585 pTX*icaADBC* were prepared, suspended in 100 mM KCl, treated with G4-DNA and hemin, and deposited on SPEs as described earlier. However, before depositing bacteria, SPEs were treated with 60 μl BG4 antibody (1:100 in 3% BSA in PBS) or a non-specific antibody (anti-mouse antibody—goat monoclonal IgG H + L, Cat No.: A-31553, Invitrogen^®^), and allowed to dry for 30 min. As controls, SPEs were treated with BG4 antibody alone or G4-DNA (5 μM). CA was carried out using 0.4 V poised potential as described earlier. Blocking experiments and CA measurements were also carried out with *S. epidermidis* 1585 pTX*icaADBC* treated with only hemin to determine the presence of endogenous G4-DNA that could interact with hemin.

### Electroanalysis of *S. epidermidis* biofilms grown directly on SPEs

We first aimed to use a wild-type *S. epidermidis* (strain 1457) and an eDNA-negative mutant (*S. epidermidis* 1457 Δ*atlE*) to determine if *S. epidermidis* biofilms are electroactive, and if this activity correlates with the presence of eDNA. We used *S. epidermidis* 1457 for this purpose because previous publications had documented formation of G4-DNA in the biofilm eDNA network formed by this strain.

Overnight cultures grown in TSB/NaCl were centrifuged (2 min at 5000 rpm) and transferred to TSB/NaCl with 5 μM hemin, adjusted to OD_600_ of 0.05, and 1.5 ml was then transferred to sterile 2 ml polystyrene cuvettes (Sarstedt AG & Co). Sterile SPEs were inserted into the solution (the connective end of SPEs was not submerged) and incubated at 150 rpm for 48 h before analysis by CV and DPV to determine the electrochemical peaks as described earlier. DPV currents measures were recorded as nano amperes per cm^2^. The average scans of three biological replicates were recorded for each voltammetric experiment. As a control experiment to separate the activity of biofilms from planktonic bacteria, planktonic cultures of both strains were grown for 48 h in TSB/NaCl + 5 μM hemin, and sterile SPEs were inserted into the culture for DPV analyses of the planktonic culture.

*Staphylococcus epidermidis* 1457 may not naturally produce a large and controllable amount of G4-DNA structures in the biofilm. To better understand how G4-DNA can contribute to EET in biofilms, we therefore used our experimental model *S. epidermidis* 1585 ± PNAG production and ± addition of G4-DNA oligos to study EET in biofilms with high or low amounts of G4-DNA structures in the extracellular matrix.

*Staphylococcus epidermidis* 1585 pTX*icaADBC* biofilms were grown in 1 ml anaerobic custom-made containers mounted directly on the SPEs. A 10 mm diameter glass vial with butyl rubber cap was cut to remove the glass bottom. The top was then attached to the SPE using non-conductive glue as described in Han *et al.* [19], with slight modifications. The container was sterilized with 70% ethanol and UV light for 15 min. BHI/NaCl (0.5 ml) with 2% xylose and/or hemin (5 μM) and G4-DNA (5 μM) was added and sparged with N_2_ gas for 2 min using sterile needles to remove oxygen before inoculation with OD_600_ 0.1 *S. epidermidis* 1585 pTX*icaADBC* overnight cultures grown in BHI/NaCl + 20 μg/ml tetracycline + 2% xylose. Control experiments were designed to determine the function of iron contained in hemin in redox transfer. We grew and treated *S. epidermidis* 1585 pTX*icaADBC* in similar conditions stated above but replaced hemin with 5 μM protoporphyrin IX (PPIX), which had similar structure with hemin but lacked iron. Electrodes were poised at +0.4 V and incubated at 37°C. The potential induced biofilm formation on the electrode, using it as an electron acceptor in the absence of oxygen. Time-based electroanalyses (CA, DPV and charge measurements) were carried out on the growing biofilms for up to 120 h using parameters described above. Immunolabelling for time-based experiments involved the use of different electrode set-ups for each time considered and immunolabeling and microscopy were subsequently conducted as described above. For nutrient replenishment experiment, 50% volume of the spent BHI/NaCl medium was removed from the set-ups and replaced at 72 h with fresh medium without hemin added. For hemin supplementation experiment, extra 5 μM hemin was added, while the medium was not changed. In both experiments of hemin and medium replenishment, electroanalyses were paused and continued after replenishments. Further experiments comparing biofilm formation on poised and unpoised electrodes in the presence and absence of oxygen were also carried out.

### Detection of G4-DNA/hemin peroxidase activity

The G4-DNA/hemin complex is also a DNAzyme with peroxidase-like activity. It could therefore also contribute to EET by transferring electrons to alternative electron acceptors, such as hydrogen peroxide (H_2_O_2_). We validated the peroxidase activity of the G4-DNA/hemin adsorbed to *S. epidermidis* 1585 pTX*icaADBC* using tyramide signal amplification (TSA). This method is commonly used in immunolabelling with horseradish peroxidase-conjugated antibody. In our assay, we visualized the location of peroxidase activity stemming from the G4-DNA/hemin DNAzyme before proceeding to test if the DNAzyme can catalyse the transfer of electrons from bacteria to H_2_O_2_.

Overnight cultures of *S. epidermidis* 1585 pTX*icaADBC* were grown in BHI/NaCl supplemented with tetracycline (20 μg/ml), and xylose (2% w/v), and contained a combination of planktonic bacteria and larger aggregates. Twenty microlitres culture was transferred to an Eppendorf tube with a cut-off pipette tip to avoid shearing of the aggregates. Aggregates were suspended in 200 μl modified MES buffer adjusted to pH 6.5. Samples were supplemented with G4-DNA (5 μM) and/or hemin (5 μM) and vortexed for (30 s) before transferring 90 μl to a coverslip with an attached Gene Frame (Thermo Scientific, AB0577) and incubating for 30 min (RT, 50 rpm shaking) to let the bacteria and aggregates adsorb before removing the liquid and replacing it with 90 μl tyramide reagent containing tyramide conjugated with Alexa Fluor 488 (1:100) (Invitrogen, B40953), 2 mM ATP (Thermo Scientific, R0441), and 0.1% hydrogen peroxide (Sigma, 216763) in modified MES buffer. Samples were then incubated for 90 min (RT, 50 rpm). Samples were washed with modified MES buffer, and stained for 30 min (RT, 50 rpm), with 90 μl of 20 μM SYTO^TM^ 60 (Invitrogen, S11342) prepared as 100× aliquots in sterile deionized water. Samples were then mounted with Prolong^TM^ Glass Antifade Mountant (Invitrogen, P3698) after a gentle wash before CLSM imaging as described earlier. Tyramide-Alexa Fluor-488 was excited at 488 nm and emission detected from <550 nm. SYTO^TM^ 60 was excited at 647 nm and emission detected at 650–700 nm. Both lasers were scanned in the same track with a beam splitter at 560 nm. Planktonic cells prepared as described earlier but not stained with Syto 60, were also used. Bright field images showed the planktonic cells during tyramide assay.

### Electrochemical detection of residual hydrogen peroxide after degradation

After confirming peroxidase activity of the G4-DNA/hemin complex, we proceeded to determine if bacteria could transfer electrons from their metabolism to H_2_O_2_ via G4-DNA/hemin. We measured the capacity for bacteria to remove H_2_O_2_, comparing *S. epidermidis* 1585 pTX*icaADBC* in the presence or absence of hemin, G4-DNA or both.

Overnight cultures of *S. epidermidis* 1585 pTX*icaADBC* were prepared as described for the TSA assay, transferred to an Eppendorf tube, diluted to OD_600_ of 1 in fresh media and supplied with G4-DNA (5 μM) and/or hemin (5 μM). Two hundred microliters culture was centrifuged (at 1118 × *g* for 2 min) and washed in same volume of 0.1 M KCl. Cells were resuspended in 200 μl modified MES buffer in 500 μl tubes and sparged with N_2_ gas for 30 s before addition of 0.1% hydrogen peroxide and incubating at 37°C with gentle shaking (40 rpm) for 5 min. After incubation, cultures were centrifuged at 1118 × *g* for 2 min, and the supernatant was collected. The supernatant (50 μl) was dropcast on sterile SPEs connected to potentiostat and CV was immediately run from +0.8 to −0.8 V at 50 mV/s to detect H_2_O_2_. Buffer containing undegraded 0.1% H_2_O_2_ served as the first control. G4-DNA (5 μM) mixed with hemin (5 μM) in 200 μl modified MES buffer containing 0.1% H_2_O_2_ were incubated anaerobically and used as a second control.

### Quantification and statistical analysis

Minimum of three independent biological replicates were used for each experiment. All statistical data analyses were done in Origin (Pro), Version 2024. Specific analyses involved the use of one-way analyses of variance (ANOVA) and Tukey’s post hoc test to assess the significance of differences between pairs of group means. Descriptive statistics involving box plots, average values, and standard deviation were also utilized in some graph plots, and this has been identified in individual figures.

## Results

### *Staphylococcus epidermidis* can interact electrochemically with hemin via eDNA

**Figure 1.**
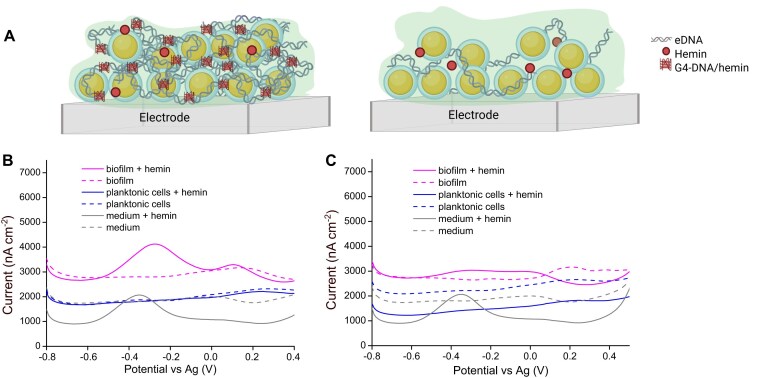
eDNA and hemin facilitate electrochemical signature of electroactive biofilms. (**A**) Illustration of biofilm formation on electrode surface with eDNA-producing and eDNA-deficient *S. epidermidis* cells. Created in BioRender. Meyer, R. (2025) https://BioRender.com/o90d703. (**B**) Differential pulse voltammogram (DPV) of biofilms and planktonic cultures of *S. epidermidis* 1457 (eDNA producing) and (**C**) *S. epidermidis* 1457 Δ*atlE* (non-eDNA producing) grown for 48 h in the presence or absence of 5 μM hemin. DPV curves are average (*n* = 3). Varying baseline currents from non-Faradaic effects are disregarded as baseline currents were not normalized in DPV curves to aid clearer visualization of the lines and peaks and DPV currents were normalised as nano amperes per cm^2^ based on electrode surface area. Sterile medium (TSB, 0.2 M NaCl, ±5 μM hemin) served as control.

Our first aim was to establish if eDNA and hemin can confer electrochemical activity in *S. epidermidis* biofilms. We used voltammetry to compare the electrochemical signatures of biofilms with high and low amounts of eDNA. Biofilms of *S. epidermidis* 1457 wildtype or an eDNA-deficient strain (*S. epidermidis* 1457 Δ*atlE)* grew directly on the surface of electrodes for 48 h in media with hemin, and voltammograms confirmed a difference between the strains (Fig. 1 and Supplementary Fig. S1). An illustration of a more robust eDNA electrode interactions formed in the biofilms of eDNA producing in comparison with eDNA deficient *S. epidermidis* is represented in Fig. 1A. In CV analyses, the wild-type strain produced slight oxidation peaks at −0.10 and +0.33 V and reduction peaks at −0.23 and −0.37 V, but these peaks were not as distinct in the eDNA-deficient mutant strain (Supplementary Fig. S1).

DPV analysis was more sensitive when applied, and sterile controls contained a peak at −0.38 V resulting from dissolved hemin (Fig. 1B). Measurements are plotted as current versus potential, which capture both Faradaic and non-Faradaic currents. Faradaic current occurs as sharp current peaks connoting specific chemical reaction resulting from redox activity, while non-Faradaic current occurs as broad background current, which arises from charging and discharging the electrical double layer at the electrode–electrolyte interface. We cultured planktonic *S. epidermidis* 1457 in media with or without hemin for 48 h and submerged the electrode to determine if planktonic bacteria could contribute to the electrochemical signal. These planktonic cultures contained no peaks—not even in samples with hemin (Fig. 1B), indicating that the bacteria removed hemin from solution, and that planktonic bacteria did not contribute to the electrochemical signature of biofilms in subsequent experiments.

In contrast to planktonic cultures, we observed two DPV peaks from biofilms grown on the electrode surface for 48 h in media with hemin (Fig. 1B). One peak (∼+0.09–0.239 V) with a midpoint at 0.109 V was attributed to the biomass, and this peak was independent of hemin in the media. Additionally, a distinct peak with a midpoint potential of −0.2802 V was only present in biofilms grown with hemin, and we ascribed this peak to the electrochemical activity of hemin embedded in the biofilm. Importantly, the position of this peak was slightly shifted relatively to the peak from dissolved hemin, indicating that hemin interacts with another component— possibly eDNA.

To test this hypothesis, we conducted the same experiment on *S. epidermidis* 1457 Δ*atlE*, which forms biofilms that contain very little eDNA (Fig. 1C). The DPV peak associated with hemin in the biofilm was much smaller for this sample, which corroborates our hypothesis that hemin interacts with eDNA to confer electrochemical activity in biofilms.

### G-quadruplex DNA/hemin complexes enable extracellular electron transport

**Figure 2.**
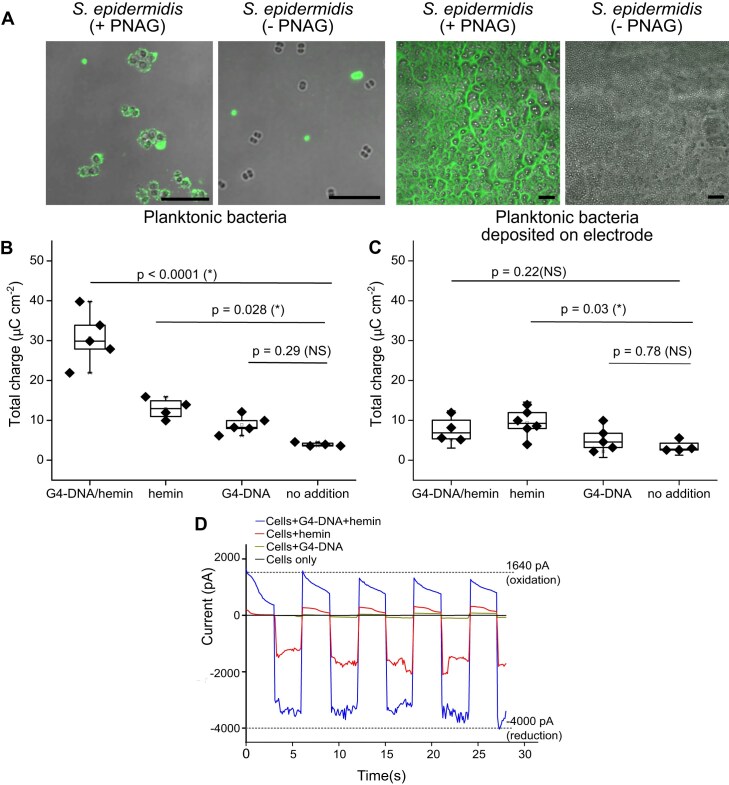
G4-DNA adsorbs on cell surface of PNAG-producing *S. epidermidis* and enables bidirectional electron flow. (**A**) Externally added G4-DNA adsorbs to the surface of planktonic *S. epidermidis* if PNAG production is induced. Planktonic *S. epidermidis* were grown with or without xylose for PNAG induction and incubated with G4-DNA for 1 h. The bacteria form a dense layer when deposited on an electrode for subsequent analysis. Confocal laser scanning microscopy (CLSM) images show bacteria (brightfield) and G4-DNA (green) visualized by fluorescence immunolabeling with antibody BG4. Scalebar = 10 μm. (**B**) Charge transfer from PNAG-induced *S. epidermidis*. Addition of G4-DNA/hemin generated significantly higher charge after 300 s at 0.4 V poised potential (total charge values included charge generated during pre-treatment time, which is both faradaic charge and capacitive charge, and recorded as microcoulombs per cm^2^). Hemin only slightly increased charge transfer if G4-DNA was absent. ∗ represents statistical significance (*P* <  .05), ANOVA and Tukey’s test, NS = not significant. (**C**) Charge transfer from PNAG-deficient *S. epidermidis* that do not bind G4-DNA. Addition of G4-DNA and hemin did not affect the total electrical charge. (**D**) G4-DNA/hemin on the surface of PNAG-induced *S. epidermidis* could release and uptake electrons bidirectionally as demonstrated by short multi-step chronoamperometric analyses (MSCA). Values are averages (*n* = 3).

To test our hypothesis that G4-DNA/hemin complexes mediate the electrochemical interaction between eDNA and hemin, we developed an experimental model using *S. epidermidis* with surface-associated G4-DNA. eDNA co-localizes with exopolysaccharides in biofilms, and we therefore used *S. epidermidis* 1585 pTX*icaADBC* [17], which has inducible production of PNAG to promote G4-DNA adsorption. We folded two different DNA oligos (c-Myc1 and c-Myc3) into G4-DNA (Supplementary Fig. S2), mixed them with *S. epidermidis*, and visualized surface-associated G4-DNA by fluorescence immunolabeling. We show that G4-DNA adsorbed primarily to PNAG-producing cells (Fig. 2A), and the G4 trimer c-Myc3 absorbed in larger quantity than c-Myc1 (Supplementary Fig. S4), resulting in higher electroactivity (Supplementary Fig. S5). We therefore chose c-Myc3 for our further analyses, and we used *S. epidermidis* 1585 pTX*icaADBC* with and without induction of PNAG production as positive and negative controls for bacteria with and without G4-DNA on the surface. *S. epidermidis* 1585 pTX*icaADBC* was used in all subsequent experiments unless specifically stated.

In an experiment to check that hemin was not toxic to the bacteria, we incubated *S. epidermidis* with or without induction of PNAG production and in the presence or absence of G4-DNA, and presence/absence of 5 μM hemin in the growth media. CFU enumeration of overnight cultures confirmed that hemin was not toxic, but it also revealed a 10-fold increase in CFU in samples with hemin. Notably, this increase in CFU only occurred in cultures with surface-adsorbed G4-DNA (Supplementary Fig. S3). Thus, hemin increased the growth yield when bacteria contained G4-DNA in the cell envelope.

Next, we grew planktonic cultures with and without induction of PNAG, added G4-DNA, hemin, or G4-DNA + hemin, and incubated for 1 h before washing and depositing the bacteria on the electrode surface for quantification of charge transfer when poised at +0.4 V versus the reference electrode, which would capture the electrochemical potential of eDNA/hemin according to our results in Fig. 1. The charge measured in this experiment depends both on the number of bacteria on the electrode surface, and their ability to transfer electrons. We therefore visualized the adsorbed bacteria and G4-DNA within the adsorbed layer. Both strains adsorbed on the electrode surface as a dense layer, but only the PNAG-producing strain contained G4-DNA (Fig. 2A). Differences in charge transfer could therefore be ascribed to differences in mechanisms for electron transfer rather than differences in biomass.

The total electrical charge measured by short chronoamperometric analysis showed that EET activity was higher for bacteria incubated with G4-DNA and hemin (Fig. 2B). In contrast, the addition of G4-DNA alone was not significantly different from the control, and the addition of hemin alone only resulted in a small increase in electrical charge. This small charge may be attributed to direct electron transfer via hemin, or by hemin bound to G4-DNA structures naturally present on the bacterial surface. When repeating the experiment on *S. epidermidis* without PNAG (and therefore minimal G4-DNA), none of the samples had a higher electrical charge than the control (Fig. 2C). These results support our conclusion that G4-DNA/hemin complexes enable extracellular electron transport.

### Surface-associated G4-DNA/hemin complexes enable bidirectional electron flow

EET facilitates both electron release (electrogenicity) and electron harvesting (electrotrophy) as reported in electroactive prokaryotes involved in biocorrosion and element cycling [6, 20]. The robustness of an EET mechanism can be shown in its ability to facilitate bidirectional electron flow [21]. We therefore sought to determine if G4-DNA/hemin in the bacterial cell envelope could convey bidirectional electron flow.

We evaluated the oxidative and reductive electron flow using short-span MSCA under oxidative (+0.4 V) and reductive (−0.4 V) potentials and quantified the current from *S. epidermidis* treated with G4-DNA, hemin, or both. We observed a larger difference in the current when poising the electrode for reduction (electron flow from the electrode to bacteria) compared to oxidation (electron flow from bacteria to the electrode) (Fig. 2D). The electron flow towards the electrode requires electron donors from bacterial metabolism, while electrons are acquired directly from the electrode surface when flowing in the opposite direction. The difference in current may therefore simply reflect the availability of electrons.

The difference in reductive and oxidative current was only observed in samples supplemented with hemin, and it was amplified when both hemin and G4-DNA were present. This lends further support to our hypothesis that G4-DNA/hemin complexes promote electron flow.

### Direct G4-DNA/hemin-electrode contact is required for electron flow

**Figure 3.**
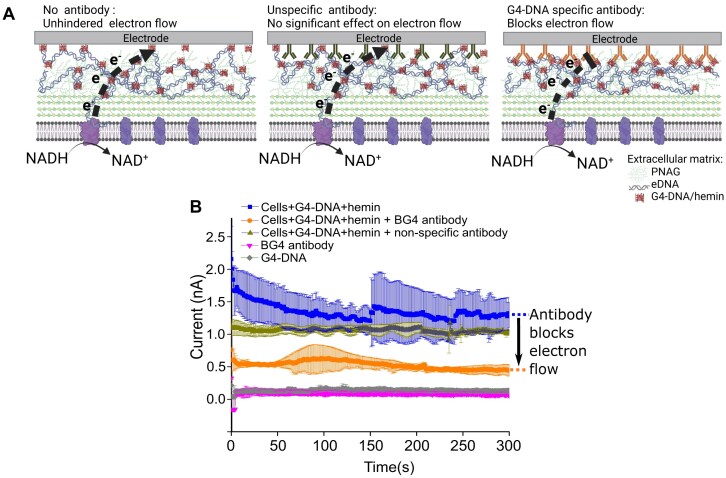
Blocking the contact between G4-DNA/hemin and the electrode surface with G4-specific antibodies disrupts EET. (**A**) An illustration of three conditions of interactions showing cells and G4-DNA/hemin directly interacting with electrode, cells interacting with electrode through unspecific antibody for G4-DNA, and cells interacting with electrode through specific G4-DNA antibody. (Created in BioRender. Meyer, R. (2025) https://BioRender.com/d14×059). (**B**) Chronoamperometric (CA) analysis of the electron flow over time (300 s) for *S. epidermidis* deposited on the electrode (mean ± SD, *n* = 3). Current was measured after 200 s pretreatment at 400 mV. ANOVA (*P* <  .05) and Tukey’s test were performed on the final values after 300 s. Coating the electrode with a G4-DNA-specific antibody reduced the electron flow from the bacteria to the electrode (indicated by black arrow).

We hypothesized that if G4-DNA/hemin complexes are essential for electron transfer, they must make direct contact with the electrode. To investigate this, we blocked the contact between G4-DNA and the electrode by coating the electrode surface with a G4-DNA specific antibody (BG4) prior to adsorption of bacteria. We then monitored electron flow by chronoamperometry. As a control, we coated the electrode with a non-specific antibody (anti-mouse IgG). The non-specific antibody did not reduce electron flow at the end of 300 s (*P* > .05) based on ANOVA and Tukey’s test, but the G4-DNA-specific antibody reduced the current by ∼1 nA (Fig. 3). Similar experiments were carried out by adding hemin to cells alone with the view of reacting with native or endogenous G4-DNAs on the bacteria surface without extra G4-DNA supplementation, and the same trend occurred (Supplementary Fig. S6). We therefore conclude that G4-DNA/hemin must be in contact with the electrode to facilitate electron flow. There is also the possibility that the G4-DNA blocking antibody could interfere with the G4-DNA/hemin structure by disrupting hemin and G4-DNA binding, which could also reduce current flow.

### Integration of G4-DNA/hemin into growing biofilms enhances electron flow

Following our analysis of planktonic bacteria, we investigated the role of G4-DNA and hemin in biofilm EET. We inoculated bacteria into the media with G4-DNA, hemin or both, and grew biofilms directly on the electrode surface (poised at +0.4 V versus reference electrode) under anoxic conditions, prompting the bacteria to use the electrode as electron acceptor. We measured the current produced throughout the 48 h of growth and subsequently visualized the biofilms by microscopy.

**Figure 4.**
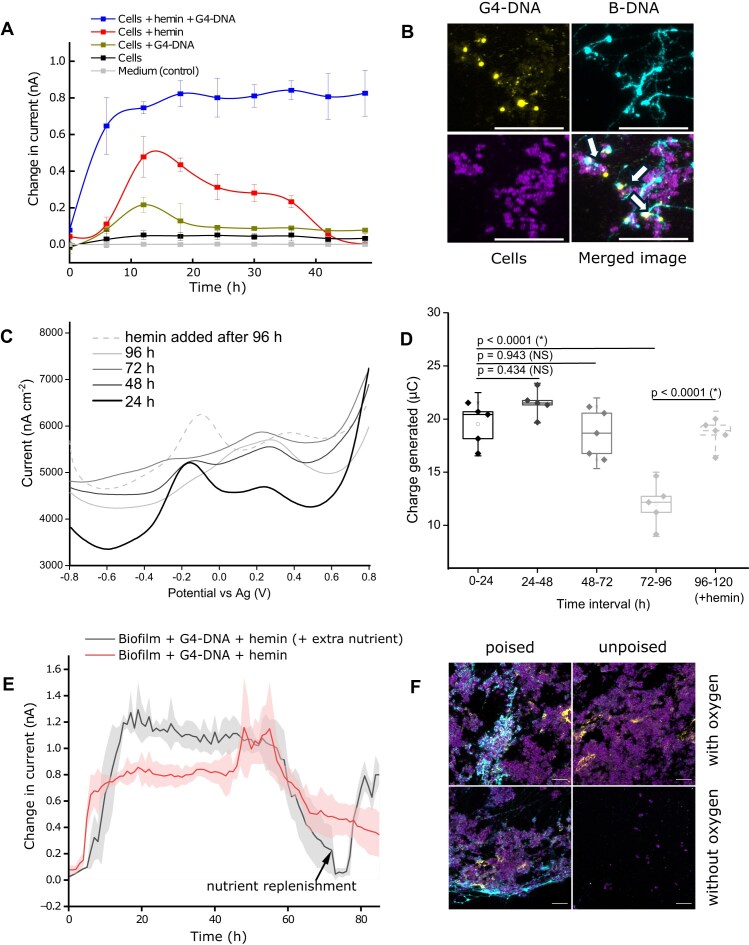
Biofilms grown with G4-DNA and hemin exhibit electroactivity for up to 48 h and sustain electroactivity for longer with hemin and nutrient replenishment. (**A**) Chronoamperometric measurements of *S. epidermidis* biofilm grown for 48 h on electrode surface poised at +0.4 V potential ± addition of G4-DNA and hemin. Current values were recorded as change in current after subtracting 8.943 nA, to give a near zero value for baseline current of the media control (**B**) CLSM images of *S. epidermidis* biofilms formed on electrode surface after 48 h growth with G4-DNA and hemin. G4-DNA forms nodular structures (indicated by white arrows) that associate with B-DNA in the biofilm. Scalebar = 20 μm. (**C**) DPV of *S. epidermidis* biofilms grown with G4-DNA and hemin on electrode surface at +0.4 V potential. The current peak decreased after 24 h but could be recovered by replenishment of hemin. Varying baseline currents from non-Faradaic effects are disregarded as baseline currents were not normalised in DPV curves to aid clearer visualisation of the lines and peaks. DPV currents were normalised as nano amperes per cm^2^ based on electrode surface area (**D**) Charge generated under different times tested from 0 to 96 h, showing decrease in electroactivity close to the 96 h age of the biofilm in comparison with 120 h old biofilm treated with additional hemin immediately after 96th hour (∗ represents significant difference between labelled experimental conditions (*P* <  .05) based on ANOVA and Tukey’s test, NS: not significant). (**E**) Current generated before and after 50% of medium was replenished at 72 h incubation time (mean ± SD, *n* = 3). (**F**) Representative CLSM images of *S. epidermidis* biofilms grown on electrodes with G4-DNA/hemin for 48 h in BHI with or without poised potentials and oxygen. Cells grown in the presence of oxygen formed biofilms with or without poised potentials. However, without oxygen, scanty biofilm biomass was formed when no potential was applied, implying that G4-DNA/hemin conferred an electroactive advantage for the biofilm. Scale bar = 10 μm.

As expected, the addition of G4-DNA alone did not promote a current from the biofilm to the electrode, while addition of hemin did. The increase in current from hemin was, however, temporary and decreased after ∼15 h. In contrast, the combination of G4-DNA and hemin resulted in a stronger and more stable current throughout the 48 h incubation (Fig. 4A). While the G4-DNA in this experiment were not generated naturally in the biofilm, these data provide proof-of-concept for the ability of G4-DNA structures to promote EET via hemin in biofilms.

Visualization of biofilms confirmed that G4-DNA was incorporated into the eDNA matrix (Supplementary Fig. S7), and some G4-DNA formed nodule-like structures (Fig. 4B and Supplementary Fig. S9). Similar nodules containing DNA were reported in electroactive *P. aeruginosa* biofilms that also performed eDNA-dependent EET(9). However, the authors did not investigate if these biofilms contained G4-DNA. G4-DNA can aggregate through π-π interactions [10], and they can also cause liquid-liquid phase separation in environments with molecular crowding [22]. The nodule-like structures may therefore reflect phase separation in the biofilm matrix. However, the mechanism of nodule-like structure formation and their specific contribution to biofilm electron transfer remains to be elucidated.

### Sustained EET requires continuous replenishment of hemin and nutrients

Our initial results indicated that EET in biofilms was stronger and more stable over time when G4-DNA/hemin was incorporated into the biofilm matrix (Fig. 4A). To test the boundaries for how stable EET is over a longer time frame, we incubated biofilms for up to 96 h in a similar experiment and carried out DPV analyses on the biofilms at 24 h intervals. The DPV peak at ∼+0.1 to +0.3 V ascribed to biomass increased from 0 to 48 h incubation and then remained stable, indicating that biofilms growth stagnated after 48 h incubation but subsequently stayed associated with the electrode (Fig. 4C). Stagnation in biofilm growth was expected as the media was not replenished, and electron donors for metabolism would become depleted. In accordance with the previous experiment, the peak around 0.09 to −0.34 V ascribed to G4-DNA/hemin increased in height during the first 48 h but subsequently decreased and disappeared at 96 h incubation (Fig. 4C). The decrease in EET at 96 h was further corroborated by measurement of charge transfer (Fig. 4D).

The decrease in EET could be caused by a decrease in metabolic activity as nutrients were depleted from the media, or it could be caused by inactivation of the G4-DNA/hemin complex due to e.g. oxidative damage or loss of Fe^2+^ or both. To test the cause of the EET decrease, we added 5 μM hemin to the biofilm to replenish hemin in the G4-DNA structures. This resulted in restoration of the G4-DNA/hemin DPV peak (Fig. 4C), demonstrating that maintaining G4-DNA/hemin-mediated EET activity requires periodic hemin replenishment. It remains to be elucidated why hemin is lost from the G4-DNA/hemin complex, and if bacteria e.g. exploit this hemin as a source of iron. Hemin is easily replenished in biofilms located in a hemin-rich environment, such as in infections.

The decrease in current could also reflect lack of nutrients, and to test this, we replenished nutrients at 72 h incubation by exchanging half of the media volume with fresh medium (without hemin). After a lag phase, the current gradually increased again, indicating metabolic activity is responsible for EET (Fig. 4E). We therefore conclude that both nutrient and hemin replenishment contribute to continuous EET in the biofilm.

These data paints a picture that *S. epidermidis* grows on the electrode by using the electrode as an electron acceptor for respiration. *Staphylococcus epidermidis* grows more slowly by fermentation in the absence of an electrode acceptor, and we therefore expected that the poised electrode would stimulate biofilm formation under anaerobic conditions. This is indeed the case (Fig. 4F). The electrode potential did not affect biofilm under aerobic conditions, but under anaerobic conditions, *S. epidermidis* formed more biofilm on the poised electrode, which further corroborates its ability to use EET to sustain metabolic activity under these conditions. We thus confirmed that cells gained a metabolic advantage under EET conditions with the aid of G4-DNA/hemin when grown without oxygen.

### Iron is essential for G4-DNA/hemin complex electroactivity

Hemin consists of PPIX coordinating a ferric iron ion (heme B) [23]. To validate the role of iron in EET observed in this study, we substituted hemin with the iron-deficient PPIX and compared electrochemical signatures based on DPV and charge generated. We grew biofilms on the electrode surface in media ± G4-DNA and supplemented with either PPIX or hemin for 48 h before DPV analysis. The DPV peak ascribed to free hemin (∼−0.35 V) was only observed in cell-free controls, indicating that free hemin had less interaction with the biofilm-covered electrodes (Supplementary Fig. S8A). Biofilms grown with G4-DNA and PPIX had only one prominent peak at ∼+0.1 to + 0.3 V, which we ascribed to biomass. In contrast, biofilms grown with G4-DNA and hemin had two peaks: one ascribed to biomass and one at ∼−0.1 to −0.3 V ascribed to the hemin/G4-DNA complex, indicating that iron is required for electroactivity. Furthermore, quantification of the charge generated from these biofilms (Supplementary Fig. S8B) showed that electroactivity required the presence of iron in hemin, and this result further corroborates iron’s importance in EET via G4-DNA/hemin.

### G4-DNA/hemin facilitates electron transfer to hydrogen peroxide

G4-DNA/hemin is a DNAzyme with peroxidase-like activity [24], and we therefore hypothesized that it also facilitates transfer of electrons from bacteria to hydrogen peroxide, which then becomes an electron acceptor for bacterial metabolism via EET.

**Figure 5.**
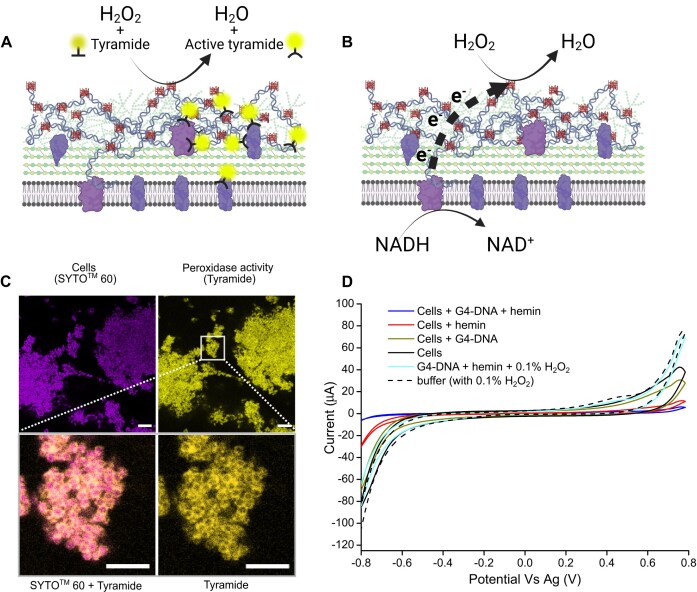
G4-DNA/hemin is an extracellular DNAzyme with peroxidase-like activity that could electrocatalytically degrade hydrogen peroxide. (**A**) Illustration showing electron flow in TSA with inactive tyramide as electron donor and H_2_O_2_ as the electron acceptor converted to H_2_O. Created in BioRender. Meyer, R. (2025) https://BioRender.com/u73k717. (**B**) Illustration showing electron flow without tyramide showing the bacterial cell as electron donor and H_2_O_2_ as the electron acceptor converted to H_2_O. (**C**) TSA shows the location of extracellular peroxidase activity (yellow) in *S. epidermidis* (magenta, SYTO60^TM^ stain) grown with xylose for PNAG induction and subsequent amendment of G4-DNA and hemin. Scale bar = 20 μm. Colours were changed in Zen Lite 3.7. Zoomed image shows that the catalytic activity is associated with the bacterial surface. Scale bar = 5 μm. (**D**) Electrocatalytic degradation of hydrogen peroxide with electrons from the bacteria. Cyclic voltammogram of electrochemical response from residual hydrogen peroxide in extracted peroxidase assay buffer after addition of cells, cells and hemin, cells and G4-DNA, or cells and G4-DNA/hemin complex.

To address this hypothesis, we first confirmed the peroxidase activity of G4-DNA/hemin in *S. epidermidis* biofilms using TSA. In this assay, the DNAzyme activates fluorescently labeled tyramide in a reaction with H_2_O_2_, leading to immobilization of fluorescent tyramide to near-by tyrosine. Biofilms amended with hemin showed a low level of extracellular peroxidase activity—likely attributable to G4-DNA/hemin complexes naturally present in the biofilm. However, biofilms amended with both G4-DNA and hemin were strongly fluorescent (Fig. 5A), confirming that the immobilized G4-DNA/hemin complex was catalytically active (Fig. 5C). We then tested if the DNAzyme can transfer electrons from the bacteria’s metabolism to H_2_O_2_ (Fig. 5B) by characterizing the electrochemical reduction of H_2_O_2_ in bacterial suspensions using CV (Fig. 5D). Addition of G4-DNA to the bacteria did not stimulate H_2_O_2_ reduction, while addition of hemin resulted in some H_2_O_2_ reduction (Supplementary Fig. S10). However, the combination of G4-DNA and hemin resulted in the highest reduction of H_2_O_2_ (Fig. 5D) evidenced by the reduced magnitude of oxidation and reduction peaks (Fig. 5D). This trend was similar to the electroactivity measured under the same conditions (Fig. 4A), indicating that G4-DNA/hemin functioned as an electron conduit from the bacteria, transferring electrons from bacteria to H_2_O_2_. These results demonstrate that extracellular G4-DNA/hemin could expand the repertoire of electron acceptors for *S. epidermidis*, and perhaps also protect the bacteria from H_2_O_2_—one of the key components in how the immune system fights pathogens [25].

## Discussion

**Figure 6.**
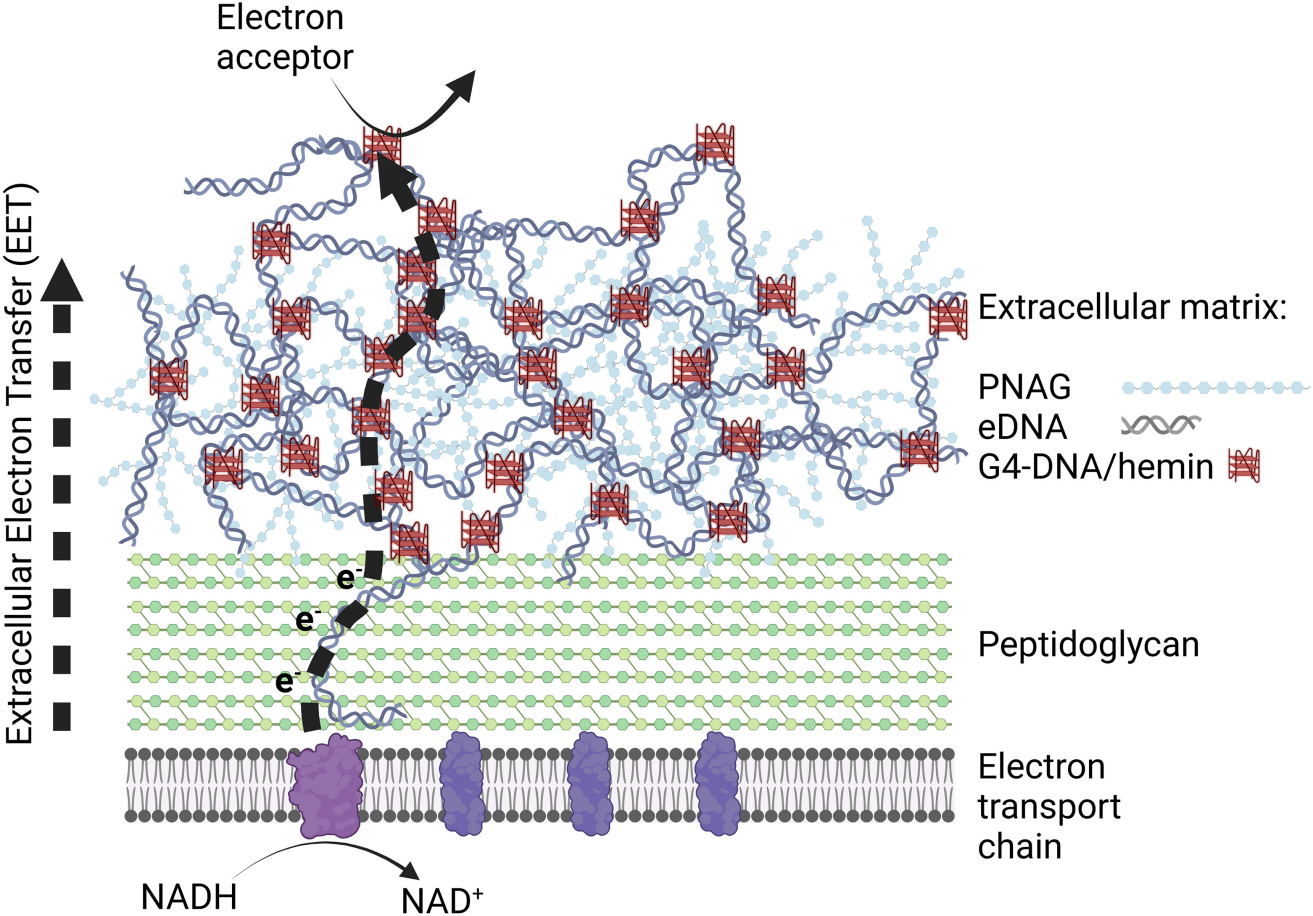
A conceptual model for the proposed involvement of G4-DNA/hemin in eDNA-mediated EET in biofilms. Created in BioRender. Meyer, R. (2025) https://BioRender.com/s74w116.

This study is the first to present a role for non-canonical DNA structures in microbial EET. We first established that EET in *S. epidermidis* biofilms requires the presence of eDNA. Since this species is known to form an eDNA network that contains G4-DNA, we hypothesized that the hemin-binding properties of G4-DNA played a role in DNA-mediated EET. The low charge transfer from soluble hemin in the absence of G4-DNA underlines the importance of the G4-DNA/hemin complex for EET (Fig. 2B and C). G4-DNA might increase charge transfer by hemin simply by facilitating its proximity to the cell, but charge transfer could also increase due to enhanced electroactivity of hemin in the G4-DNA/hemin complex [26, 27] caused by the π–π stacking in G-quartets and the planar porphyrin structure of hemin [28]. Previous studies have shown that G4-DNA structures are an integral part of the eDNA network in staphylococcal biofilms [2], and here we propose a mechanistic model for EET in biofilms via G4-DNA/hemin complexes (Fig. 6).

Electrons from metabolic processes enter the electron transport chain of *S. epidermidis* when NADH and FADH_2_ donate electrons to NADH dehydrogenase (NDH) or succinate dehydrogenase (SDH) [29]. NDH and SDH both transfer electrons to soluble menaquinone which transfers electrons to cytochromes and eventually to oxygen. In EET, electrons are diverted out of the cell. Based on the redox potential of hemin (∼-0.2 to −0.4 V versus Ag/AgCl reference electrode), electrons are probably intercepted at NDH or SDH. NDH contributes to generating the proton motive force, and diverting electrons from NDH to hemin instead of menaquinol will still result in ATP production.

How might the electrons be transferred from NDH on the surface of the cell membrane to G4-DNA/hemin outside of the cell wall? G4-DNA structures are too wide to pass through the 2 nm pores of peptidoglycan, and the bacteria must use another mechanism to move electrons up through the peptidoglycan, which spans tens of nanometers. Electron flow across the cell envelope differs in Gram-positive and Gram-negative bacteria. While many electroactive Gram-negative bacteria use multiheme cytochromes [30], these are uncommon in Gram-positive bacteria such as *Staphylococcus*. Instead, Gram-positive bacteria tend to secrete soluble redox mediators, such as nicotinaminde adenine dinucleotide in *Bacillus subtilis* [31], and flavins and quinones in *Bacillus cereus*, *Rhodococcus ruber* [32], and *Lactiplantibacillus plantarum* [33]. *Listeria monocytogenes* immobilizes secreted redox mediators in specific extracellular proteins (PplA) that span the peptidoglycan [34]. Another component that could facilitate electron transfer across the peptidoglycan are secreted conductive polymers. For example, *B. subtilis* forms long structures of poly γ-glutamic acid that make up a conductive biofilm matrix [35]. The exact mechanism for how electrons cross the peptidoglycan in *S. epidermidis* remains to be investigated.

Hemin might perform a similar role as secreted redox mediators, but rather than being secreted, pathogenic bacteria can acquire hemin from blood. At the site of an infection, hemin enters the blood as a result of hemolysis or tissue damage, reaching levels of up to 25 μM [36]. While hemin binds to G4-DNA with high specificity, it can also interact with other biomolecules and thereby become immobilized in the cell wall through such interactions. Hemin possesses a hydrophobic ring with a positively charged iron core, enabling interaction with proteins [37], lipids [38], and membrane vesicles [39]. Due to its positive charge and planar geometry, hemin might interact with lipo- or wall-teichoic acids, which are negatively charged and constitute up to 50% of the cell wall mass [40]. It is thus possible that hemin contributes to electron transfer across the peptidoglycan, either as a soluble or immobilized redox mediator.

### Comparison with DNA-mediated EET in other bacteria

DNA can be conductive in the right conformation and chemical environment. Canonical B-DNA allows electrical charge transfer between π orbitals of closely arranged base pairs [41], and DNA structures are being considered for programmable nanoelectronics [42] and batteries [43]. Notably, G4-DNA is more conductive than B-DNA because the stacked guanine tetrads facilitate π–π interactions [44]. Multiple G4-DNA can also stack into conductive G-wires reaching up to 100 nm [45]. This situates G4-DNA as a likely candidate for EET. DNA is the one matrix component that most biofilms have in common, and G4-DNA structures have so far been reported in laboratory grown biofilms of *P. aeruginosa*(3) and *S. epidermidis*, in implant-associated *S. aureus* infections,[2] and in dental plaque [46]. It is thus likely that G4-DNA is present in many biofilms, but its relevance was overlooked until now.

EET in biofilms can involve direct electron transfer via conductive biomolecules or indirect electron transfer using soluble redox-active molecules [5, 6, 21]. Direct electron transfer via eDNA has been shown in *Shewanella*[47], and a combination of direct and indirect electron transfer via eDNA and phenazines was reported for *P. aeruginosa*[9]. With the vast abundance of iron porphyrins in nature, we propose that G4-DNA and hemin provide a generic mechanism for EET that is conceptually similar to what was described for *P. aeruginosa*.

We show in this study that *S. epidermidis*, which is not perceived as an electroactive microorganism, does convey eDNA-dependent electron transfer in biofilms (Fig. 1). Moreover, EET could be stimulated and maintained when providing additional G4-DNA and hemin to the biofilms (Fig. 3A). We ascribe the stable EET to the immobilization of hemin in the biofilm and integration of electroactive G4-DNA/hemin complexes in the wider network of eDNA. Furthermore, hemin in G4-DNA/hemin complexes may be better protected from oxidative damage compared to free hemin [48], and thus provide a more stable EET.

The use of DNA for EET requires a mechanism by which electrons can be transferred to and from the conductive DNA. While we propose that hemin is key to achieving such electron transfer, there are also other DNA-binding redox-active molecules, such as flavins and phenazines, which can increase DNA conductivity after being bound to DNA [9, 49]. Similarly, chemosynthetic redox-active molecules such as methylene blue and monomethine cyanine dyes have also been demonstrated to bind to G4-DNA [50, 51]. The principle for eDNA-mediated EET described in this study may therefore go far beyond the specific mechanism described here.

### Biological implications of G4-DNA/hemin-mediated EET

In nature, bacteria use EET to access insoluble electron acceptors or to reach electron acceptors (e.g. oxygen) at tens of micrometers from the cell [9]. Thus, EET can support aerobic respiration by bacteria in anoxic microenvironments—a capability that is highly relevant for pathogens that cause biofilm infections. As new research shows that extracellular G4-DNA structures are abundant in biofilms, we propose that EET is more widely used than previously assumed and that EET can play a role in the pathogenicity of biofilms.

In addition to mediating EET, we show that G4-DNA/hemin also facilitate reduction of H_2_O_2_ with electrons from the bacteria. Host cells produce H_2_O_2_ in response to an infection, [25, 52] and bacteria could use G4-DNA/hemin to defend against H_2_O_2_ in the infectious microenvironment, and even exploit H_2_O_2_ as an electron acceptor for respiration.

Another host defense mechanism is to limit the access of bacteria to iron [53]. Pathogens have therefore developed mechanisms to obtain iron from heme or hemin. For example, *S. aureus* contains near iron transporter (NEAT) domain proteins and Isd (iron-regulated surface determinant) for uptake of entire heme molecules from hemoproteins such as hemoglobin [54, 55]. *Staphylococcus epidermidis* lacks the machinery for heme uptake but utilizes siderophores [56], and acquisition of iron from immobilized hemin could explain the increase in growth yield caused by hemin when bacteria contained surface-associated G4-DNA (Supplementary Fig. S3). Hence, immobilization of hemin in G4-DNA/hemin complexes on the cell envelope may serve as a mechanism for accessing iron in the infectious microenvironment. Natural heme containing molecules such as catalases and cytochromes bo and aa3 are present in *S. epidermidis*. However, these heme-containing molecules are protein based, and their hemes are locked up in a configuration within the protein making it not easily accessible for EET. The need for free heme molecules externally tethered to the bacterial cell envelope remains the ideal condition for outward electron flow that defines EET as an energy regulatory mechanism.

## Conclusion

In summary, this study reveals a novel mechanism by which bacteria can perform EET through G4-DNA/hemin complexes in their biofilm matrix. This finding has multiple implications: it demonstrates a previously unknown role for non-canonical DNA structures in bacterial metabolism, reveals a potentially widespread mechanism for bacterial survival in oxygen-limited environments, and suggests a dual benefit in pathogenesis where the same mechanism could support both respiration and defense against oxidative stress. The discovery that G4-DNA structures can serve as electrical conduits when complexed with hemin not only advances our understanding of bacterial physiology but also opens new possibilities for applications in bioelectrochemical systems and synthetic biology. As we continue to uncover the diverse roles of DNA structures in bacterial biofilms, this work provides a foundation for understanding how bacteria have evolved to use nucleic acid structures beyond their genetic function.

## Supplementary Material

gkaf790_Supplemental_File

## Data Availability

The data underlying this article will be shared on reasonable request to the corresponding author.
