# Supplementary material for: Hemin-binding DNA structures on the surface of bacteria promote extracellular electron transfer
Source: Nucleic Acids Res. 2025 Aug 21;53(15):gkaf790. doi: 10.1093/nar/gkaf790 (PMC12370626; doi:10.1093/nar/gkaf790)
Supplement: gkaf790_Supplemental_File [file gkaf790_supplemental_file.pdf]

Hemin-binding DNA structures on the surface of bacteria promote extracellular electron transfer

Obinna M. Ajunwa[1,2], Gabriel Antonio S. Minero[1], Sissel D. Jensen[1], Rikke L. Meyer[1,3]*

[1] Interdisciplinary Nanoscience Center (iNANO), Aarhus University, Gustav Wieds Vej 14, 8000 Aarhus, Denmark
[2] Center for Electromicrobiology, Department of Biology, Aarhus University, Ny Munkegade 114, DK-8000 Aarhus C, Denmark
[3] Department of Biology, Aarhus University, Ny Munkegade 114, 8000 Aarhus, Denmark

* To whom correspondence should be addressed. Email: rikke.meyer@inano.au.dk

SUPPLEMENTARY MATERIAL

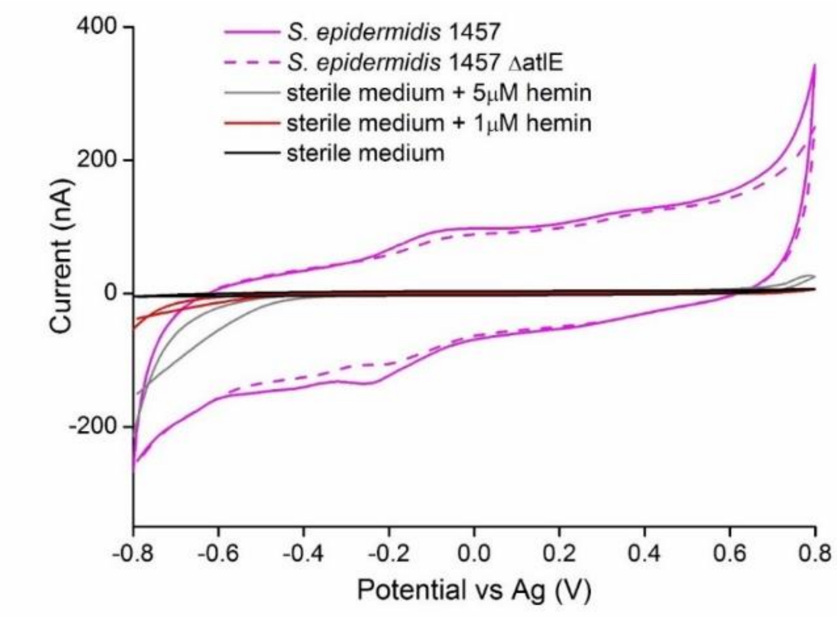

Figure S1. Cyclic Voltammogram (CV) of biofilms formed by *S. epidermidis* 1457 (eDNA producing) and *S. epidermidis* 1457 Δ*atlE* (autolysin deficient and low eDNA producing) when grown with 5 μM hemin for 48 h. Sterile TSB with 0.2 M NaCl medium and sterile medium (+1 μM and +5 μM hemin) served as controls.

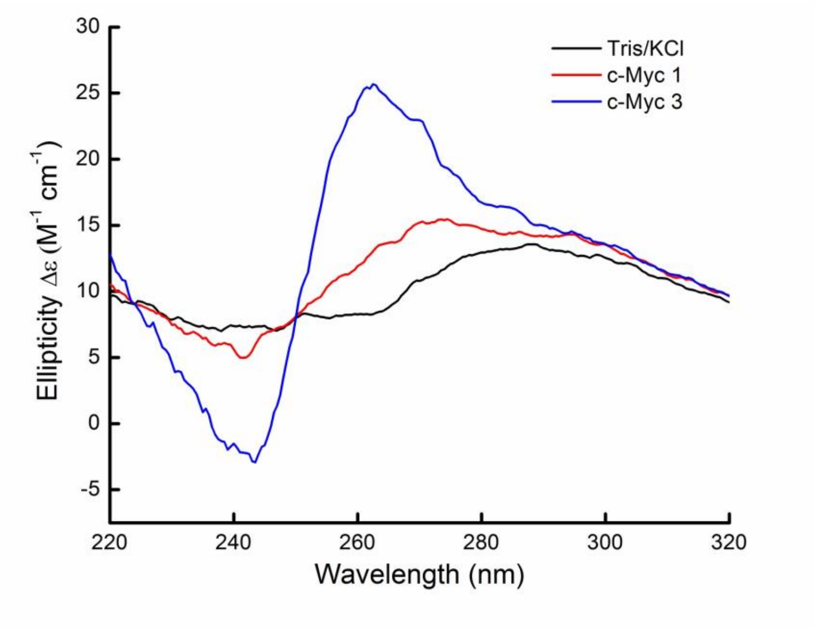

Figure S2. Circular dichroism confirms the structure of G4-DNA oligonucleotides

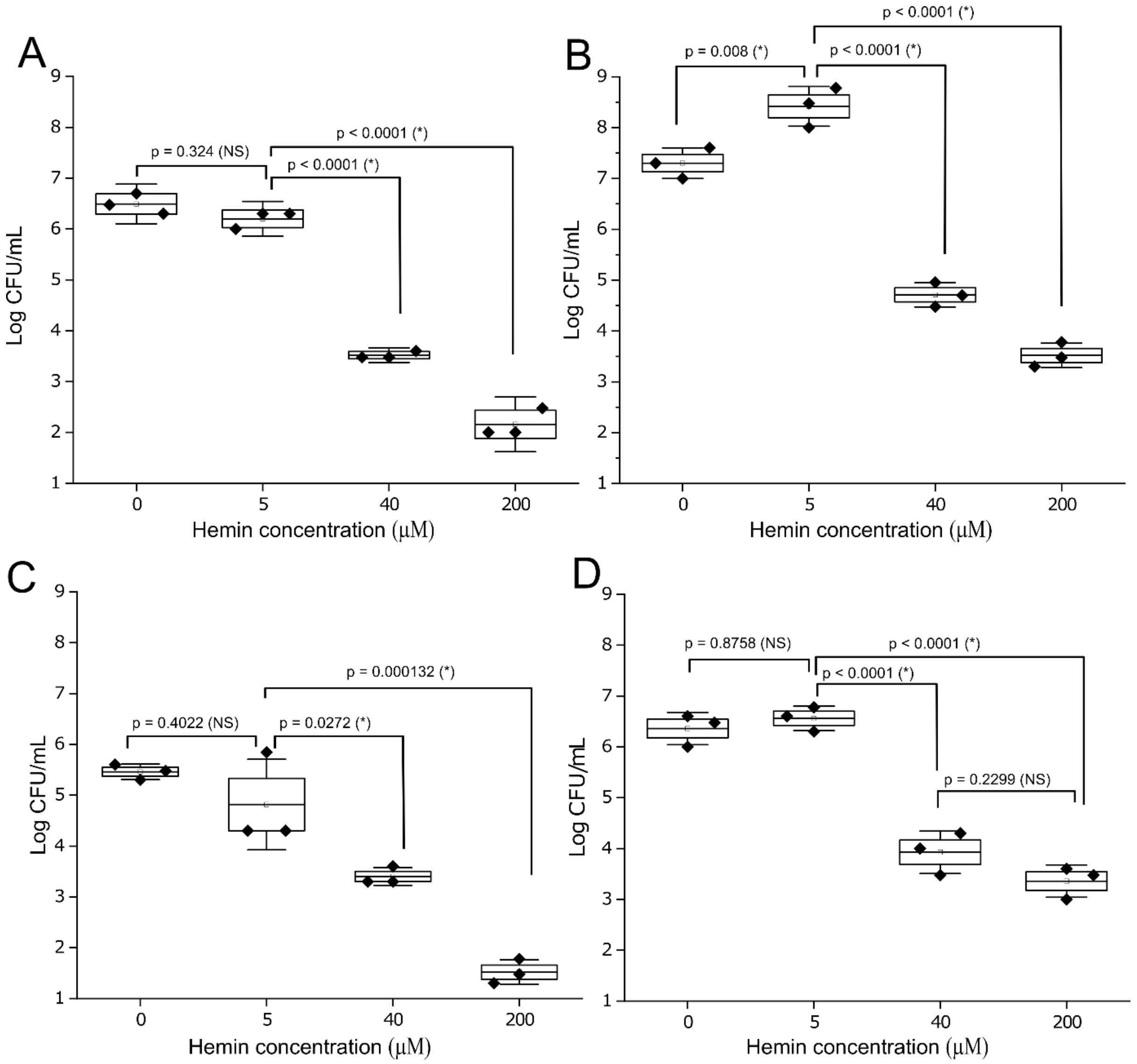

Figure S3. Cell viability and tolerance tests of planktonic cultures of *S. epidermidis* 1585 pTX*icaADBC* grown in varying concentrations of hemin with or without PNAG induction and G4-DNA (c-Myc3, 5 μM) : (A) PNAG -, G4-DNA + (B) PNAG +,G4-DNA + (C) PNAG - , G4-DNA - (D) PNAG +, G4-DNA –. ∗ represents statistical significance ($p < 0.05$), ANOVA and Tukey's test, NS= Not significant.

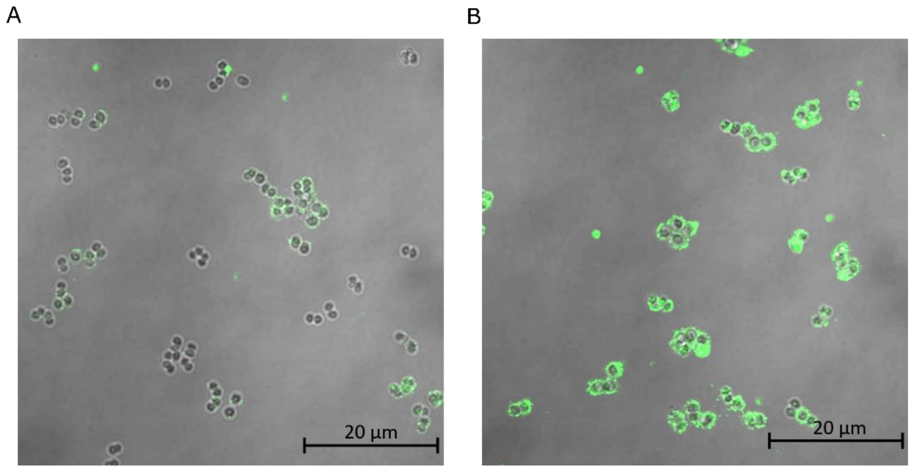

Figure S4. **Adsorption of c-Myc1 (A) and c-Myc3 (B) to the surface of *S. epidermidis* with induced PNAG production.** G4-DNA was detected by immunolabeling (antibody BG4, green). More G4-DNA was detected on the bacterial surface when using c-Myc3, and this oligo was therefore used in our subsequent experiments.

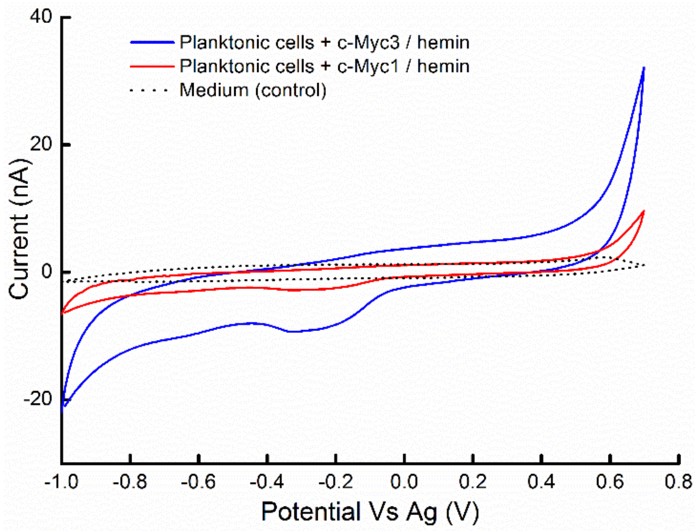

Figure S5. Cyclic Voltammogram (CV) shows that bacteria with c-Myc3 are more electroactive. Planktonic *S. epidermidis* were grown in TSB/NaCl with xylose (for PNAG production) and treated with 5 µM G4-DNA and 5 µM hemin before adsorption to the electrode and measurement of CV.

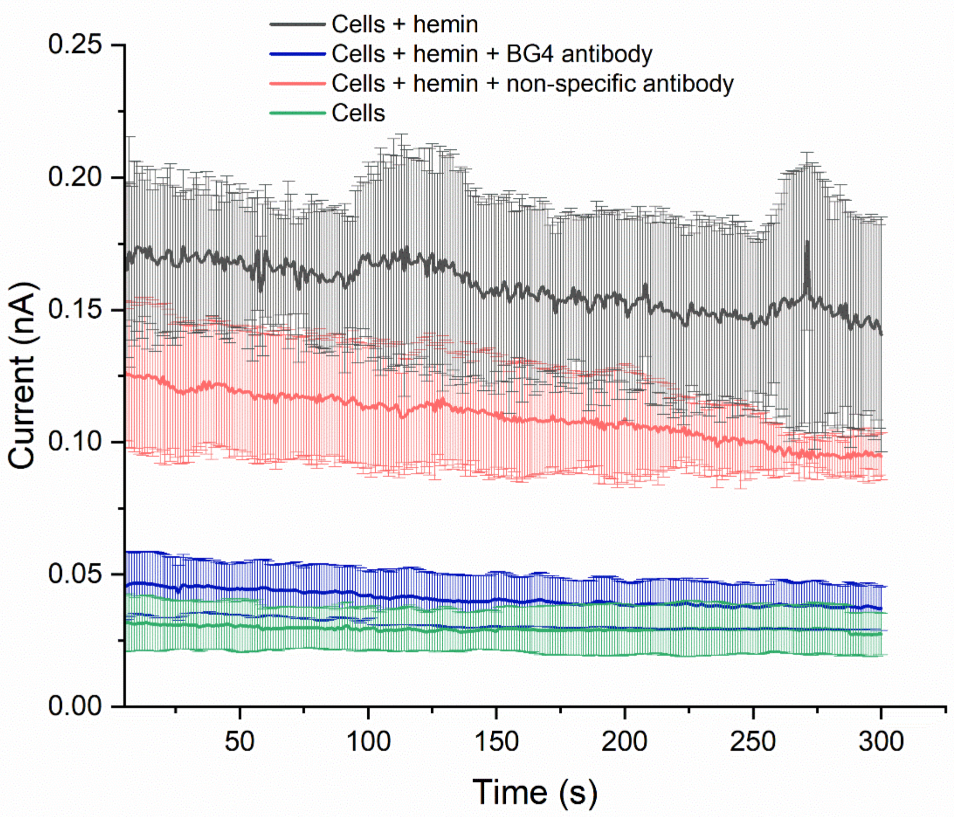

Figure S6. Chronoamperometric (CA) traces of electron flow from hemin treated *S. epidermidis* with endogenous G4s interacting with antibodies on electrode. Cells augmented with only hemin showed more current reduction when exposed to adsorbed G4-specific antibodies on electrode than when exposed to non-specific antibodies. This proves that natural G4s on the surface of cells interact differently with the G4-specific and non G4-specific antibodies. Untreated cells served as the control. Values are mean +/- SD of n=3.

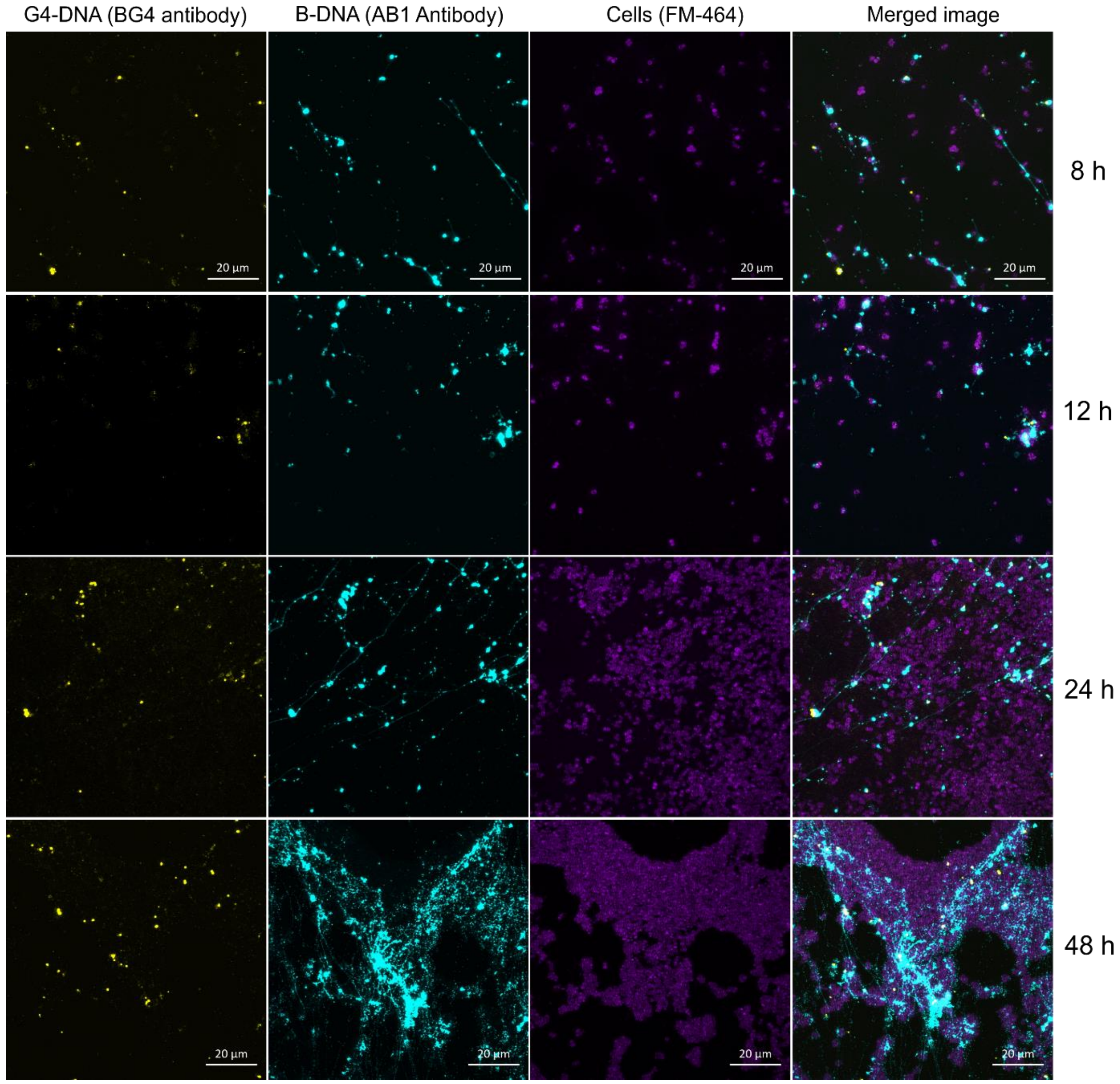

Figure S7. Externally added G4-DNA are incorporated into the biofilm matrix as biofilms grow. Biofilms were grown directly in the electrode poised at +0.4 V vs. the reference electrode. G4-DNA was supplied in the growth medium. CLSM images of biofilms from 8 h to 48 h show that G4-DNA was incorporated into the biofilm. Cells are show in magenta (stained with FM4-64, B-DNA is show in cyan (labeled with antibody AB1), and G4-DNA is shown in yellow (labeled with antibody BG4).

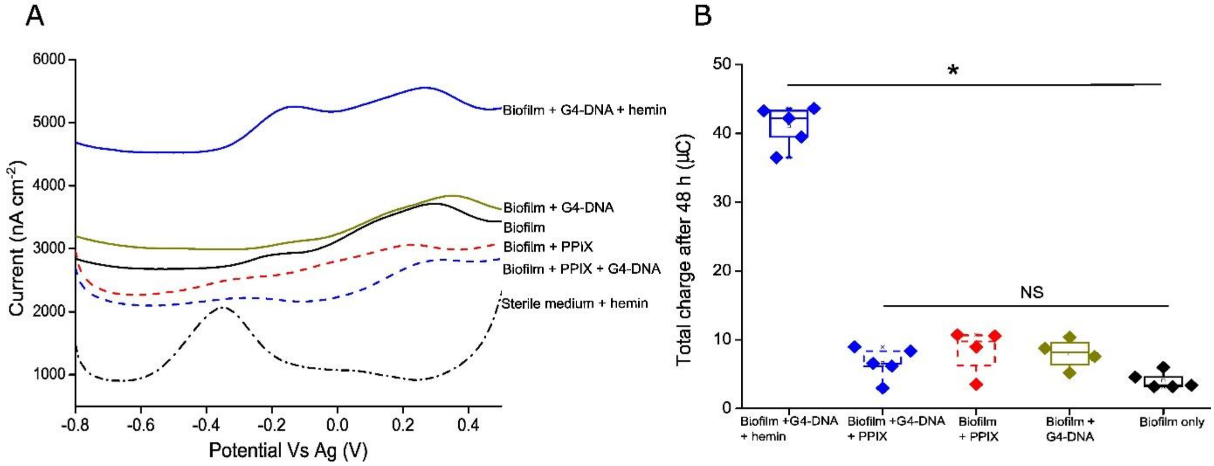

Figure S8. Biofilms grown with G4-DNA and hemin facilitate EET in an iron-dependent fashion. Comparative electroactivity of 48 h old PNAG-induced *S. epidermidis* biofilms supplemented with G4-DNA and either hemin or the iron deficient PPIX. (A) DPV shows PPIX treated biofilms lack the characteristic negative potential peak (-0.3 V to +0.1 V) associated with G4-DNA/hemin interactions. Varying baseline currents from non-Faradaic effects are disregarded as baseline currents were not normalized in DPV curves to aid clearer visualization of the lines and peaks and DPV currents were normalized as nano amperes per cm^2 based on electrode surface area (B) Total electric charge generated from biofilms also confirm the requirement of iron porphyrin to facilitate charge transfer (∗ represents significant difference ($p < 0.05$) based on ANOVA and Tukey's test, NS: Not significant).

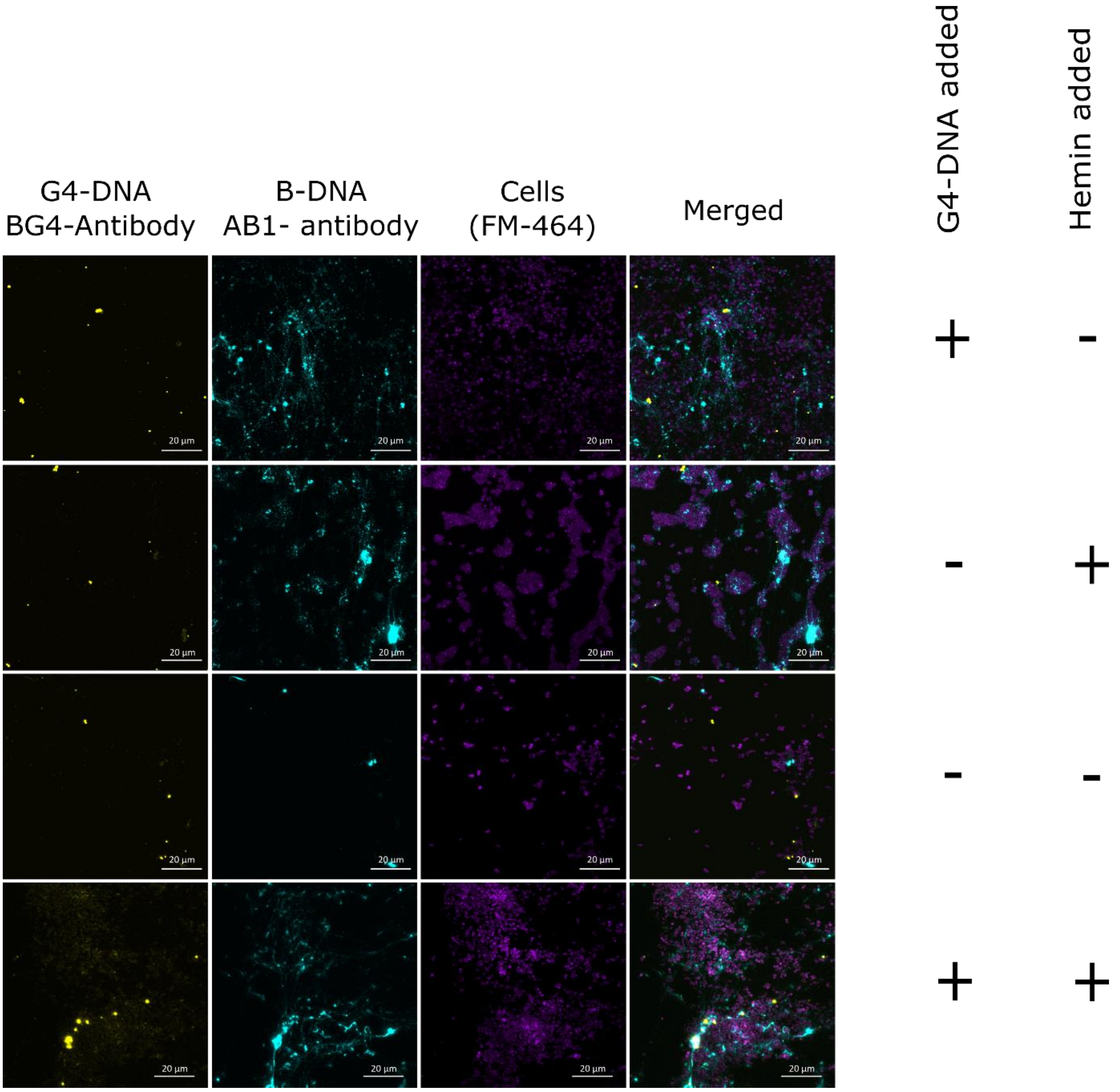

Figure S9. Representative CLSM images of *S. epidermidis* 1585 pTX*ica* biofilms grown on electrodes for 48 h in TSB with or without G4-DNA and hemin. In samples with both G4-DNA and hemin, the extracellular DNA forms nodule-like structures rich in G4-DNA that associate with the B-DNA network in the biofilm.

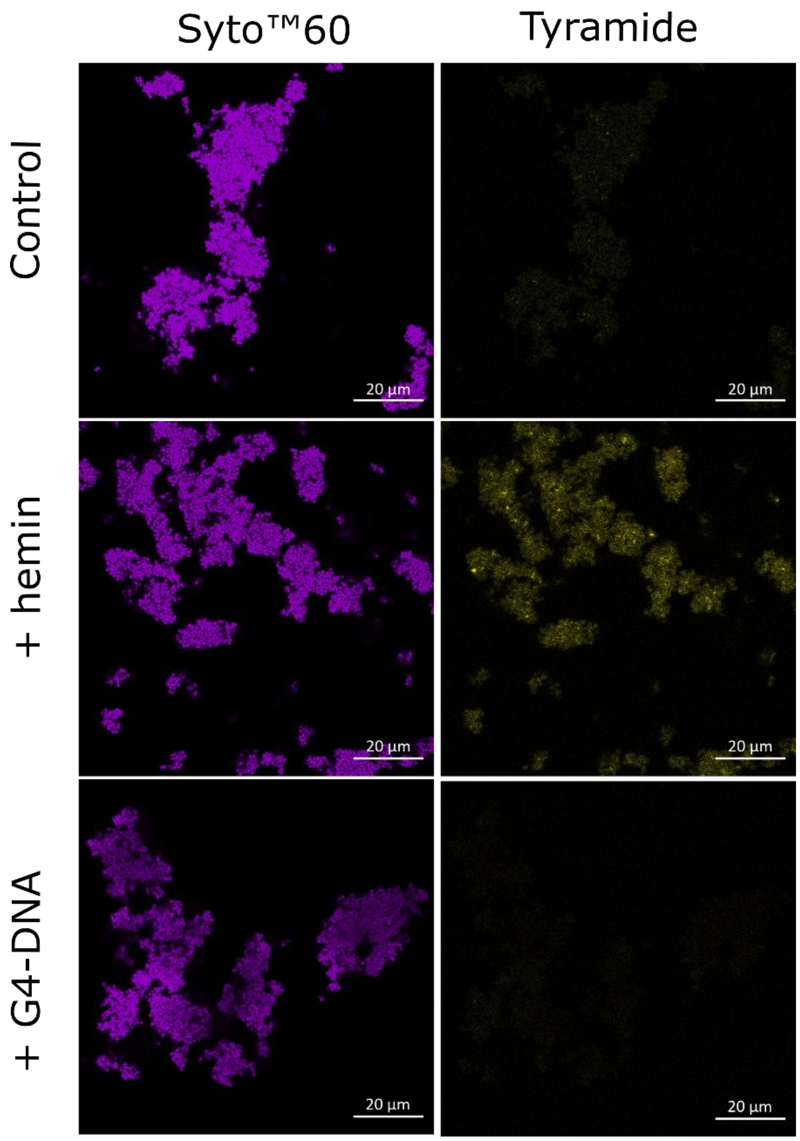

Figure S10. Lack of G4-DNA/hemin on the bacteria results in low peroxidase-like activity. Tyramide signal amplification (yellow) shows low signals around bacteria (SYTO 60^{TM}, magenta) that were grown with either hemin or G4-DNA but not both. Scale bar = 20 µm.
